# Arterial stiffness is related to a higher risk of cardiovascular events in patients with pseudoxanthoma elasticum (PXE)

**DOI:** 10.1177/1358863X251394284

**Published:** 2026-01-21

**Authors:** Melanie Haverkamp, Pim A de Jong, Frank LJ Visseren, Wilko Spiering

**Affiliations:** 1 Department of Vascular Medicine and Endocrinology, University Medical Center Utrecht, Utrecht University, Utrecht, The Netherlands; 2 Department of Radiology, University Medical Center Utrecht, Utrecht University, Utrecht, The Netherlands

**Keywords:** arterial stiffness, cardiovascular risk, pulse wave analysis, pulse wave velocity, pseudoxanthoma elasticum (PXE), rare vascular disease

## Abstract

**Background:** Pseudoxanthoma elasticum (PXE) is a rare disease caused by pathogenic *ABCC6* variants, leading to arterial calcifications and increased cardiovascular risk. Validated intermediate endpoints are needed to evaluate cardiovascular risk-reducing therapies in PXE. This prospective cohort study investigates the relationship between arterial stiffness and cardiovascular events in patients with PXE. **Methods:** This prospective cohort study obtained patients from the Dutch University Medical Center Utrecht Expertise Center for PXE. Arterial stiffness was measured with carotid-femoral pulse wave velocity (cfPWV) and the augmentation index (AIx). Cardiovascular endpoints were cardiovascular death, cerebrovascular and coronary events, and peripheral artery interventions. Cox proportional hazard models analyzed associations between arterial stiffness and cardiovascular events, adjusting for confounders. **Results:** Among 390 patients (mean age 51 ± 15 years, 60% women), 45 cardiovascular events occurred during a median follow up of 6.1 years (IQR 3.2; 9.2). A 1-m/s higher cfPWV was related to an increased risk of cardiovascular events (hazard ratio [HR]: 1.28; 95% CI 1.06–1.53). The effect of cfPWV depends on age (*p*-value = 0.03), with a lower cardiovascular risk HR at older age (HR age at 40 years: 1.51; 95% CI 1.11–1.97 to HR age at 60 years: 1.12; 95% CI 1.00–1.26). A 10% higher AIx at baseline was related to future cardiovascular events (HR 1.46; 95% CI 1.10–1.95). No significant interaction between the AIx and age was found. **Conclusion:** This prospective cohort study shows that arterial stiffness, measured by cfPWV and AIx, is independently associated with increased cardiovascular risk in PXE. Measures of arterial stiffness could be explored as intermediate endpoints in trials evaluating cardiovascular risk-reducing therapies in PXE.

## Background

Pseudoxanthoma elasticum (PXE, #OMIM264800) is a rare, inherited disease caused by pathogenic genetic variants in the *ABCC6* gene.^1,2^ These pathogenic variants are associated with lower levels of plasma inorganic pyrophosphate (PPi), which is a natural inhibitor of calcification.^3^ The decreased levels of PPi are thought to result in ectopic calcifications in the Bruch’s membrane of the retina, the skin, and the arteries.^4^ These calcifications are associated with severe vision loss, visible skin abnormalities, and vascular diseases like peripheral artery disease (PAD) and cerebrovascular disease.^5–8^ Ischemic strokes and PAD are more frequently seen in the PXE population than in the general population.^8,9^ The increased risk of cardiovascular events is associated with total arterial calcification (TAC) volume on a total body computed tomography (CT) scan. Patients with PXE with higher calcification volumes have a higher risk of developing a new cardiovascular event.^10^

Arterial calcification is mainly found in the medial arterial wall of patients with PXE.^11^ Medial arterial calcification causes stiffening of the arterial wall.^12^ The reference standard to measure arterial stiffness is the carotid-femoral pulse wave velocity (cfPWV), which mainly reflects the stiffness of the aortic pathway.^13,14^ A cfPWV of more than 10 m/s is considered elevated.^14,15^ An alternative method for assessing arterial stiffness is measuring the augmentation index (AIx) with pulse wave analysis (PWA), which measures the stiffness of peripheral arteries.^15^

Arterial stiffness is more pronounced in patients with PXE than in the general population.^16^ In the general population, arterial stiffness, measured by PWV, is associated with an increased risk of cardiovascular events and improves cardiovascular risk classifications adjusted for cardiovascular risk factors.^16–18^ The relationship between PWV and cardiovascular events was not only found in the general population, but also in patients with end-stage renal disease, essential hypertension, type 2 diabetes mellitus (DM), and elderly individuals.^19^ However, the relationship between arterial stiffness and the occurrence of cardiovascular events in patients with PXE is not yet known.^16^

Arterial stiffness might be an intermediate endpoint for cardiovascular events in patients with PXE. As PXE is a rare disease, a validated intermediate cardiovascular endpoint could be used in clinical trials evaluating treatments directed at reducing cardiovascular risk. However, before arterial stiffness can be considered as an intermediate endpoint, it is essential to establish whether it is associated with cardiovascular events in PXE. This prospective cohort study aims to evaluate the association between arterial stiffness and the occurrence of cardiovascular events in patients with PXE.

## Methods

### Study population

Patients were derived from the Dutch UMC Utrecht Expertise Center for Pseudoxanthoma elasticum (UECP). All patients attending the UECP are asked to participate in the Dutch PXE registry: a prospective cohort study using pseudonymized data from regular care visits. The study was approved by the institutional review board of the UMC Utrecht, and all patients gave their written informed consent. All patients who attended the UECP between 2008 and October 2024, with a definitive or probable PXE diagnosis according to the Plomp criteria, were included (supplemental Table S1). Patients were enrolled in the cohort at their first visit to the UECP. As arterial stiffness measurements have been part of the standard of care since 2013, follow up started from the first arterial stiffness measurements. Patients without arterial stiffness measurements at baseline (due to logistical reasons or being under 18 years old) were excluded. This manuscript was prepared in accordance with the STROBE reporting guidelines.^20^

At the baseline visit, information on medical history, physical examination, and laboratory measurements was retrieved. The systolic and diastolic blood pressures are the mean of three consecutive measurements. Low-density lipoprotein cholesterol (LDL-c) levels were calculated using the Friedewald formula.^21^ The estimated glomerular filtration rate (eGFR) was calculated using the Chronic Kidney Disease Epidemiology Collaboration (CKD-EPI) formula.^22^ During regular care visits, patients undergo extensive vascular examinations, including measurements of arterial stiffness, a treadmill test, a 6-minute walk test, carotid intima–media thickness, and a total body CT scan.

### Arterial stiffness measurements

The cfPWV and PWA for measurement of the AIx are measured by two trained research nurses in the supine position after patients have rested for at least 5 minutes. The cfPWV measures the speed at which the forward pressure wave travels along the aorta to the femoral artery and mainly reflects the stiffness of the pathway of the aorta. The stiffer the arteries become, the faster the pressure wave travels, which causes an increased cfPWV.^14,15^ An alternative method for assessing arterial stiffness is measuring AIx with PWA. The AIx is the difference between the first and second systolic peaks caused by the wave reflection of peripheral arteries. It is expressed as a percentage of the pulse pressure.^15^ The stiffer the arteries become, the faster the reflected wave returns from the peripheral arteries, which results in a higher AIx. The measurements were performed using the SphygmoCor device (AtCor Medical, Sydney) until 2018 and the SphygmoCor XCEL (AtCor Medical, Sydney) device from 2019 onwards.^23^ A comparison of cfPWV and AIx measurements between the two devices showed no significant differences (Table S2). The PWA is performed with brachial cuff placement. The parameters measured for the PWA were aortic systolic and diastolic blood pressure, pulse pressure, and augmentation pressure. The AIx was calculated by dividing the augmentation pressure by the pulse pressure and expressed as a percentage. The AIx was corrected for a heart rate of 75 bpm according to a generally accepted correction method.^24^

For the cfPWV, the carotid and femoral pulses were recorded by applanation tonometry when using the SphygmoCor device. The SphygmoCor XCEL device records the carotid pulse with applanation tonometry, and the femoral pulse was measured using a blood pressure cuff around the upper thigh. Before July 2019, the travel distance (TD) was assessed along the body using the indirect distance or subtracted method (TD = [suprasternal notch – femoral pulse site] – [suprasternal notch – carotid pulse site]). After this period, the distances were calculated using the direct distances method (TD = carotid pulse site – femoral pulse site). The indirect distances were recalculated into direct distances using a standardized equation (TD_direct_ = 0.45*X_indirect_ + 0.21 × height + 0.08) to enable comparison between patients with different measurement methods.^25^ Transit times are the automatically measured difference in time between the arrival of the pulse wave in the carotid and femoral arteries. The cfPWV was then calculated using the following equation: TD_direct_*0.8/transit time.^14,26^

### Outcomes

The primary outcome is the occurrence of cardiovascular events during follow up, which is a composite score of cardiovascular death, cerebrovascular events (transient ischemic attack [TIA], amaurosis fugax, and stroke), coronary events (myocardial infarction, elective percutaneous coronary intervention [PCI], and coronary artery bypass graft [CABG]), and peripheral artery interventions (peripheral artery stent or surgery, amputation of toe or foot or limb). If patients experienced multiple events during follow up, only the first event was used in the analysis.

Cardiovascular death is defined as death resulting from cardiovascular diseases, which are identified through linkage with death registers using *International Classification of Diseases*, Tenth Revision (ICD-10) codes (I00–I99 – Diseases of the circulatory system). At each follow-up visit at the UECP, patients were systematically asked whether any cardiovascular events had occurred. To ensure up-to-date information, follow-up questionnaires were sent to all participants; in cases of nonresponse, additional contact attempts were made by telephone. In case of an event, correspondence from the attending hospital was requested to verify the diagnosis. New cardiovascular events were scored according to the endpoints by two physicians independently of each other.

### Statistical analyses

Baseline characteristics are presented and stratified according to PWV tertiles. Baseline characteristics were presented as mean ± SD for normally distributed continuous variables or median with IQR for nonnormally distributed variables. Categorical variables were summarized as counts and percentages. Additionally, a baseline table was stratified according to normal and high PWV values based on individual, age-adjusted reference PWV thresholds. Expected PWV values were calculated for each patient using validated formulas based on their age group and mean arterial pressure.^27^ Patients with an observed PWV value above the expected value were categorized as having a high PWV value. Missing data (⩽ 5% for all variables) were imputed by single imputation using predictive mean matching.

Cox-proportional hazard models were used to calculate hazard ratios and corresponding 95% CIs for the relationship between arterial stiffness and the occurrence of cardiovascular events, adjusted for potential confounders. For simpler clinical interpretation, age was centered around the mean age of the study population. This means that the hazard ratios show the risk for a patient at the mean age of the study population (51 years). Hazard ratios and 95% CIs were calculated for cfPWV as a continuous variable per 1-m/s increase, and AIx as a continuous variable per 10% increase. Three models were made. First, crude models were made. Second, models were adjusted for age and sex. Third, models were adjusted for DM, kidney function, smoking, systolic blood pressure, antihypertensive therapy, and cardiovascular history. Potential confounders were selected based on previous literature and clinical judgment. The selected potential confounders represent well-known cardiovascular risk factors that are associated with both arterial stiffness and cardiovascular events. Smoking, DM, hypertension, and chronic kidney disease are known to have an impact on arterial stiffness due to arterial calcification, inflammation, and changes in the arterial wall.^28–30^ Moreover, effect modification by age and cardiovascular history was evaluated by including interaction terms in the fully adjusted models. It was hypothesized that age could be an effect modifier for arterial stiffness based on previous literature.^18^ As cardiovascular history was an effect modifier in the relationship between TAC and cardiovascular events, its role was also tested in the relationship between arterial stiffness and cardiovascular events.^10^

The proportional hazard assumption, which was assessed visually on plotted Schoenfeld residuals, was not violated. No significant evidence of nonlinearity between cfPWV, AIx, age, and cardiovascular events was found by plotting martingale residuals and formally by adding age as a restricted cubic spline function to the models.

As an exploratory analysis, the relationship between arterial stiffness on the occurrence of cerebrovascular events was assessed and adjusted for age, sex, DM, kidney function, smoking, systolic blood pressure, and cardiovascular history. Another sensitivity analysis was conducted that only included patients with PXE who were compound heterozygous or homozygous for two (likely) pathogenic variants in the *ABCC6* gene. Moreover, a sensitivity analysis for the relationship between arterial stiffness and acute cardiovascular events was performed. Acute cardiovascular events were defined as cerebrovascular events (stroke, TIA, and amaurosis fugax), myocardial infarctions, and cardiovascular deaths. This analysis was additionally adjusted for age, sex, DM, kidney function, smoking, systolic blood pressure, and cardiovascular history.

All statistical analyses were performed using R statistical software, version 4.4.2 (R Foundation for Statistical Computing), and for all analyses *p* < 0.05 was considered statistically significant.

## Results

A total of 494 individuals with PXE were initially identified. After applying diagnostic criteria and assessing arterial stiffness, 390 individuals with definitive or probable PXE and available arterial stiffness measurements were included in the study. The mean age was 51 ± 15 years, and 60% of patients were women. There were 59 (15%) patients with a history of cardiovascular events. The baseline characteristics according to cfPWV values are displayed in Table 1. Patients with higher cfPWV values were older and more often men than patients with a normal cfPWV. Moreover, patients with a higher cfPWV have more cardiovascular risk factors, such as DM, smoking, higher pulse pressure, and a greater carotid intima–media thickness. They also have a higher prevalence of cardiovascular disease history and are more often treated with blood pressure-lowering medication, lipid-lowering medication, and antithrombotic medication (Table 1). When patients are stratified based on their age-expected cfPWV values, 327 (84%) patients have a higher than expected cfPWV (Table S3).

**Table 1.** Patient characteristics according to tertiles of pulse wave velocity (PWV).

	PWV Tertile 1	PWV Tertile 2	PWV Tertile 3
	4.3–8.8 m/s	8.9–11.0 m/s	11.1–22.5 m/s
	*n* = 130	*n* = 130	*n* = 130
Age (years)	38 ± 14	55 ± 10	61 ± 8
Female sex	93 (72)	72 (55)	68 (52)
Diabetes mellitus	2 (2)	9 (7)	15 (12)
Smoking status			
Never	65 (50)	48 (37)	41 (32)
Former	51 (39)	60 (46)	73 (56)
Current	14 (11)	22 (17)	16 (12)
History of CVD			
CAD	4 (3)	8 (6)	12 (9)
CeVD	2 (2)	2 (2)	10 (8)
PAI	2 (2)	2 (2)	8 (6)
> 1 location	0 (0)	1 (1)	8 (6)
Antihypertensive treatment	12 (9)	31 (24)	55 (42)
Lipid-lowering treatment	18 (14)	38 (29)	71 (55)
Antithrombotic therapy	7 (5)	24 (19)	43 (33)
Glucose-lowering therapy	2 (2)	8 (6)	11 (9)
BMI (kg/m^2^)	24.5 ± 5.1	27.1 ± 4.7	26.9 ± 4.1
SBP (mmHg)	121 ± 15	133 ± 15	145 ± 19
DBP (mmHg)	72 ± 9	76 ± 8	80 ± 9
Total cholesterol (mmol/L)	4.8 ± 1	5.2 ± 1.2	5.2 ± 1.2
LDL-cholesterol (mmol/L)	2.7 [2.3–3.4]	2.9 [2.3–3.6]	2.8 [2.2–3.7]
HDL-cholesterol (mmol/L)	1.5 ± 0.4	1.5 ± 0.4	1.5 ± 0.4
eGFR (mL/min/1.73 m^2^)	108 ± 18	93 ± 13	87 ± 13
Pulse pressure (mmHg)	39 ± 10	47 ± 14	56 ± 16
AIx (%)	22 ± 15	32 ± 13	33 ± 11
Carotid IMT (μm)	595 [543–690]	731 [646–822]	811 [723–907]

Data are presented as *n* (%), mean ± SD, or median [IQR].

AIx, augmentation index; BMI, body mass index; CAD, coronary artery disease; CeVD, cerebrovascular disease; CVD, cardiovascular disease; DBP, diastolic blood pressure; eGFR, estimated glomerular filtration rate with the Chronic Kidney Disease Epidemiology Collaboration formula; HDL, high-density lipoprotein; IMT, intima–media thickness; LDL, low-density lipoprotein; PAI, peripheral artery disease with arterial interventions; SBP, systolic blood pressure.

### Arterial stiffness and occurrence of cardiovascular events

The median follow-up duration was 6.1 years [IQR 3.2; 9.2] with a total follow up of 2,386 person-years. During follow up, 45 cardiovascular events occurred (incidence rate 1.9 per 100 person-years). The incidence rates increased across cfPWV tertiles, ranging from 0.5 in the lowest tertile to 3.3 in the highest tertile. Cerebrovascular events were the most frequently observed (*n* = 32, 71%). In eight patients (18%), a cardiac event occurred, and four patients (9%) experienced a peripheral artery intervention. One patient (2%) died after a cardiovascular event. The cerebrovascular events consisted of 19 cerebral infarctions, 11 TIAs, and two cases of amaurosis fugax. Among the cardiac events, there were five myocardial infarctions, one CABG, and two elective PCI procedures.

#### Carotid-femoral pulse wave velocity (cfPWV) and cardiovascular risk

**Figure 1.**
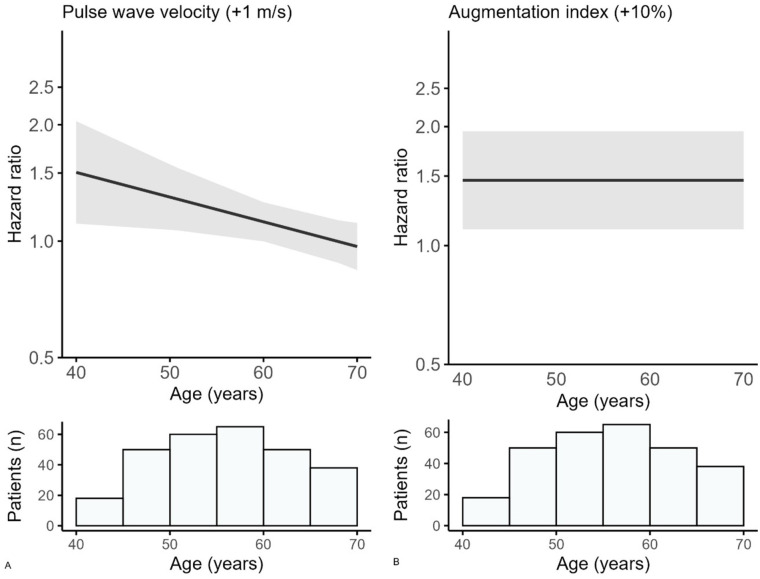
Relation between **(A)** carotid-femoral pulse wave velocity and **(B)** augmentation index, and risk of cardiovascular events across age.

The observed cardiovascular risk was higher across tertiles of cfPWV (supplemental Figure S1). A higher cfPWV (per 1 m/s) was related to a higher risk for cardiovascular events (HR 1.28; 95% CI 1.06–1.54 for mean age) (Table 2). A 1-SD (3.0 m/s) increase in cfPWV was related to cardiovascular events (HR 2.08; 95% CI 1.20–3.58 for mean age). The relation between cfPWV and cardiovascular risk decreases with increasing age (*p*-value for interaction 0.02) (Table 2). At age 40 years, the HR is 1.51 (95% CI 1.11–2.04), decreasing to 1.30 (95% CI 1.07–1.58) at age 50, and 1.12 (95% CI 1.00–1.26) at age 60 years. At age 69 years, there is no relation between cfPWV and cardiovascular risk (HR 0.98; 95% CI 0.86–1.12) (Figure 1).

**Table 2.** Relationship between arterial stiffness and risk of cardiovascular events (*N* = 390 patients).

Total events, *n* (%)	45 (11.5)
Events per 100 py	1.9
Pulse wave velocity (+ 1 m/s), HR [95% CI]^a^	
Crude model	1.16 [1.07–1.24]
Model 1	1.22 [1.03–1.44]
Model 2	1.28 [1.06–1.54]
Augmentation index (+ 10%), HR [95% CI]	
Crude model	1.50 [1.18–1.90]
Model 1	1.37 [1.04–1.82]
Model 2	1.46 [1.10–1.95]

a Hazard ratios are estimated at a mean age of 51 years.

Model 1 was adjusted for age and sex.

Model 2 was adjusted for age, sex, DM, eGFR, smoking, SBP, antihypertensive treatment, and CV history. Model 2 for PWV was additionally adjusted for the interaction between age and PWV.

CV, cardiovascular; DM, diabetes mellitus; eGFR, estimated glomerular filtration rate; PWV, pulse wave velocity; py, person-years; SBP, systolic blood pressure.

#### Augmentation index (AIx) and cardiovascular risk

The observed cardiovascular risk was higher across tertiles of AIx and is shown in Figure S2. In the adjusted model, there was a statistically significant relation between a 10% higher AIx and the risk of cardiovascular events (HR 1.46; 95% CI 1.10–1.95) (Table 2). A 1-SD increase in AIx (14%) was related to cardiovascular events with an HR of 1.67 (95% CI 1.14–2.46). No significant interaction was found for age in the relation between AIx and cardiovascular risk (*p*-value for interaction 0.11).

### Arterial stiffness and occurrence of cerebrovascular events

In total, 33 cerebrovascular events occurred during follow up. A 1-m/s higher cfPWV was related to a higher risk for cerebrovascular events, although the relationship was not statistically significant (HR 1.17; 95% CI 0.94–1.46). A 10% increase in the AIx was related to a higher risk of cerebrovascular events (HR 1.40; 95% CI 1.00–1.96) (Table 3).

**Table 3.** Relationship between arterial stiffness and risk of cerebrovascular events (*N* = 390 patients).

Total events, *n* (%)	33 (8.5)
Events per 100 py	1.4
Pulse wave velocity (+ 1 m/s), HR [95% CI]^a^	
Crude model	1.17 [1.08–1.27]
Model 1	1.19 [0.97–1.45]
Model 2	1.17 [0.94–1.46]
Augmentation index (+ 10%), HR [95% CI]	
Crude model	1.56 [1.18–2.06]
Model 1	1.42 [1.02–1.96]
Model 2	1.40 [1.00–1.96]

a Hazard ratios are estimated at a mean age of 51 years.

Model 1 was adjusted for age and sex.

Model 2 was adjusted for age, sex, DM, eGFR, smoking, SBP, and CV history. Model 2 for PWV was additionally adjusted for the interaction between age and PWV.

CV, cardiovascular; DM, diabetes mellitus; eGFR, estimated glomerular filtration rate; PWV, pulse wave velocity; py, person-years; SBP, systolic blood pressure

### Arterial stiffness and occurrence of cardiovascular events in patients with compound heterozygous or homozygous *ABCC6* variants

In total, 310 patients (79%) were identified as either compound heterozygous or homozygous for two (likely) pathogenic variants in the *ABCC6* gene. The median follow-up duration was 6.4 years [IQR 3.2; 9.4] with a total follow up of 1939 person-years. During follow up, 35 cardiovascular events occurred (incidence rate 2.01 per 100 person-years). A higher cfPWV (per 1 m/s) was related to a higher risk for cardiovascular events (HR 1.26; 95% CI 1.00–1.59 for mean age). The relation between cfPWV and cardiovascular risk decreases with increasing age (*p*-value for interaction 0.05). For the AIx, a relationship was found between a 10% higher AIx and the risk of cardiovascular events (HR 1.47; 95% CI 1.04–2.08) (Table S4).

### Arterial stiffness and occurrence of acute cardiovascular events

In 40 patients, a first, acute cardiovascular event occurred during follow up. The most common events during follow up were 32 cerebrovascular events (including two cases of amaurosis fugax, 11 TIAs, and 19 ischemic strokes), six myocardial infarctions, and two cardiovascular deaths. The median follow-up duration was 6.2 years [IQR 3.2; 9.4] with a total follow up of 2414 person-years. A higher cfPWV (per 1 m/s) was related to a higher risk for cardiovascular events (HR 1.26; 95% CI 1.03–1.54) (Table S5). The relation between cfPWV and cardiovascular risk decreases with increasing age (*p*-value for interaction 0.05). For the AIx, a relationship was found between a 10% higher AIx and the risk of cardiovascular events (HR 1.65; 95% CI 1.22–2.22) (Table S5).

## Discussion

In this prospective cohort study, it is shown that for every 10% increase in the AIx, the risk of a cardiovascular event significantly increases by 46%, independent of other cardiovascular risk factors. Also, a 1-m/s increase in cfPWV was related to a higher risk of cardiovascular events. This relationship changed with age for cfPWV, with a stronger association observed in younger patients.

Arterial stiffness is a well-known cardiovascular risk factor in the general population. Studies on the relationship between AIx and cardiovascular events show inconsistent results. In healthy participants, the AIx showed no significant relationship with cardiovascular events (HR 0.91; 95% CI 0.77–1.07).^17^ Another population-based cohort showed a minimal association between major cardiovascular events and the AIx (HR 1.05; 95% CI 1.00–1.11).^31^ However, two meta-analyses on the relationship between AIx and cardiovascular events did show significant associations between AIx and cardiovascular events (HR 1.18; 95% CI 1.09–1.27 and HR 1.32; 95% CI 1.09–1.59).^32,33^ The differences in risks may arise from varying study populations, as the meta-analyses mainly included patients with end-stage renal disease, strokes, and coronary artery disease (CAD). In our study, each 10% increase in AIx was associated with a 46% higher risk of cardiovascular disease, which was even stronger than the 18% and 32% risk increase reported in the meta-analyses of patients with CKD, CAD, and hypertension.

The higher risk of AIx in PXE may suggest that AIx is a more sensitive marker of cardiovascular risk in PXE. We hypothesized that this stronger association may be explained by the location of the arterial calcifications in PXE.^9^ In patients with PXE, arterial calcifications are mainly found in the carotid siphon and peripheral arteries, like the arms and the legs.^9^ These calcifications are located in the medial layer of the arterial wall, which leads to stiffer peripheral arteries.^16^ As pulse wave reflection originates mainly from the lower extremities, stiffer peripheral arteries amplify the pulse wave reflection, which results in a higher AIx.^34^ This is also observed in patients with PAD, in whom higher AIx values are found as well.^35^ As peripheral calcifications are a dominant problem in patients with PXE, the AIx may reflect PXE pathophysiology better than in other populations.

The reference standard for assessing arterial stiffness is the cfPWV, which reflects aortic stiffness. In a large prospective cohort study of healthy participants, patients with a 1-SD higher cfPWV had a 48% higher risk of a cardiovascular event.^17^ These results differ from our study, where a 1-m/s increase in cfPWV was associated with a 28% higher risk of cardiovascular events. This discrepancy may be explained by differences in study populations or by the way cfPWV was expressed (per 1-SD vs per 1-m/s increase). In another large meta-analysis of 17 prospective studies, it was found that a 1-m/s increase in cfPWV increased the cardiovascular risk by 10–40%, which is more consistent with the increased risk found in our study.^36^ Also, in a meta-analysis involving patients with DM, the pooled HR was 1.15 (95% CI 1.07–1.24), which is comparable to our findings, possibly due to overlapping calcification patterns that are found in patients with DM and PXE.^11,12,37^

The effect of cfPWV on cardiovascular events depended on age in our study. Among younger patients, cfPWV showed a stronger association with cardiovascular events than among older patients. In a recent meta-analysis involving almost 18,000 individuals, including cohorts with patients with known diseases and population-based cohorts, it was also shown that the effect of cfPWV was more pronounced in younger individuals.^18^ In our study, the effect of cfPWV diminished with age, and, from the age of 69 years, the CV risk was no longer present. However, in the meta-analysis with 18,000 individuals, the risk of cfPWV on cardiovascular events was still observed in older patients, although lower than in younger patients. It is not entirely clear why the effect of cfPWV decreases at older ages in PXE. We can speculate about several possible explanations. First, we hypothesized that the diminished effect at older ages may be due to other risk factors, such as TAC and traditional cardiovascular risk factors, playing a more significant role at older ages, which might suppress the effect of cfPWV alone. Recently, it was shown that TAC volume on a total body CT scan in PXE patients is related to cardiovascular events.^10^ In PXE, arterial calcifications start developing at a young age, mostly in the peripheral arteries, and become more widespread at older ages, which could reduce the effect of the cfPWV alone.^9^ Second, we hypothesized that the AIx may be a more reliable marker in patients with PXE as it reflects stiffness of the peripheral arteries. Though the AIx measures the stiffness of the peripheral arteries, the cfPWV mainly measures the stiffness of the aorta. As the aorta is less affected in PXE patients, the AIx might better reflect cardiovascular risk in PXE. Third, cfPWV may reach a plateau at a certain age, beyond which it no longer discriminates cardiovascular risk in PXE. If arterial stiffness is already so pronounced, further increases in PWV could add only a little information.

Our results indicate that increased arterial stiffness, assessed by the AIx and cfPWV, is related to the risk of incident cardiovascular events in patients with PXE. Measures of arterial stiffness may be used as an intermediate endpoint in future clinical trials evaluating medical treatment directed towards lowering cardiovascular risk in patients with PXE. PXE is a rare disease, and therefore clinical studies are of a limited sample size, not allowing the occurrence of ‘hard’ cardiovascular endpoints to be studied. Therefore, intermediate vascular endpoints are important to evaluate potential treatments. No treatment for reducing cardiovascular risk in PXE is available yet, other than treatment of conventional atherosclerotic risk factors such as a healthy lifestyle, lowering blood pressure, and treating plasma cholesterol. Several novel therapies are currently being investigated, and effects on the vasculature could be evaluated with intermediate vascular endpoints.^38–40^ Recently, it was shown that calcification volume on CT might be a useful intermediate endpoint, but arterial stiffness is much easier to measure in clinical practice, is less expensive than measuring calcification volume, and no radiation is needed. The measurement of arterial stiffness is a simple, noninvasive, and reproducible method. This supports arterial stiffness as a potential future intermediate endpoint in clinical trials evaluating preventive treatment for PXE. However, whether arterial stiffness is a valid intermediate outcome remains to be established, as its responsiveness to treatment and eventual clinical events in PXE have not yet been demonstrated.

One of the strengths of our study is the relatively large prospective cohort of patients with PXE, given the rarity of the disease. Additionally, comprehensive information was available for all patients regarding diagnosis and vascular assessments, and the cardiovascular events were investigated thoroughly. Another strength is that arterial stiffness was measured and examined by two different techniques, which resulted in a good representation of arterial stiffness in patients with PXE. Moreover, to our knowledge, this is the first study that investigates the relationship between arterial stiffness and cardiovascular events in a PXE population.

Some limitations need to be considered. First, we only include baseline parameters according to arterial stiffness measurements. These values might have changed during follow up and were not taken into account. Second, the number of events in the youngest patients is low, which limits the ability to reliably estimate HRs in this group and results in wide confidence intervals. Therefore, the HRs for the youngest patients should be interpreted carefully. Third, not all patients were examined with the same device for the cfPWV and AIx measurements. As the new device was only recently implemented, there was insufficient power to analyze only patients assessed with this new device. However, our comparison of absolute measurement values and validation studies showed no reason to believe that the newer device showed different values for the cfPWV and AIx.^41–43^

## Conclusion

Arterial stiffness, measured by cfPWV and AIx, is independently related to a higher risk of cardiovascular events in patients with PXE. Measures of arterial stiffness may be tested as an intermediate endpoint in clinical studies evaluating the effect of interventions on the risk of cardiovascular events in patients with PXE.

## Supplemental Material

sj-docx-1-vmj-10.1177_1358863X251394284 – Supplemental material for Arterial stiffness is related to a higher risk of cardiovascular events in patients with pseudoxanthoma elasticum (PXE) Supplemental material, sj-docx-1-vmj-10.1177_1358863X251394284 for Arterial stiffness is related to a higher risk of cardiovascular events in patients with pseudoxanthoma elasticum (PXE) by Melanie Haverkamp, Pim A de Jong, Frank LJ Visseren and Wilko Spiering in Vascular Medicine
