# Supplementary material for: Arterial stiffness is related to a higher risk of cardiovascular events in patients with pseudoxanthoma elasticum (PXE)
Source: Vasc Med. 2026 Jan 21;31(1):19–27. doi: 10.1177/1358863X251394284 (PMC12901643; doi:10.1177/1358863X251394284)
Supplement: sj-docx-1-vmj-10.1177_1358863X251394284 – Supplemental material for Arterial stiffness is related to a higher risk of cardiovascular events in patients with pseudoxanthoma elasticum (PXE) [file sj-docx-1-vmj-10.1177_1358863X251394284.docx]

# SUPPLEMENTARY MATERIAL

| Table S1: Plomp criteria | | |
| --- | --- | --- |
| Skin |  |  |
|  | Major criteria |  |
|  |  | Yellowish papules and/or plaques on the lateral side of the neck and/or flexural areas of the body |
|  |  | Increase of morphologically altered elastin with fragmentation, clumping, and calcification of elastic fibers in a skin biopsy taken from clinically affected skin |
| Eyes |  |  |
|  | Major criteria |  |
|  |  | Peau d’orange of the retina |
|  |  | One or more angioid streaks, each at least as long as one disk diameter |
|  | Minor criteria |  |
|  |  | One angioid streak shorter than one disk diameter |
|  |  | One or more ‘comets’ in the retina |
|  |  | One or more ‘wing signs’ in the retina |
| Genetics |  |  |
|  | Major criteria |  |
|  |  | A pathogenic variant of both alleles of the *ABCC6* gene |
|  |  | A first-degree relative (parent, sib, child) who meets independently the diagnostic criteria for definitive PXE |
|  | Minor criteria |  |
|  |  | A pathogenic variant of one allele of the *ABCC6* gene |

Definitive diagnosis: Two (or more) major criteria not belonging to the same category. Probable diagnosis: Two major eye or two major skin criteria or one major criterion and one (or more) minor criteria not belonging to the same category as the major criterion. Possible diagnosis: A single major criterion or one or more minor criteria. (Plomp et al. 2010)

Table S2: Comparison according to measurement device

|  | SphygmoCor device  N=261 | SphygmoCor XCEL  N=129 | Median difference [95% CI] |
| --- | --- | --- | --- |
| cfPWV (m/s) | 10.0 [4.4] | 9.7 [2.5] | 0.3 [-0.2;1.0] |
| AIx@75bpm (%) | 29.3 [14.5] | 24.6 [17.2] | 4.7 [-0.8;4.8] |

Data are presented as median [interquartile range]. Abbreviations: cfPWV: carotid-femoral pulse wave velocity; AIx@75bpm: Augmentation index adjusted for a heart rate of 75 beats per minute.

| Table S3: Patient characteristics according to cfPWV | | |
| --- | --- | --- |
|  | Normal cfPWV* | High cfPWV* |
|  |  |  |
|  | N = 63 | N = 327 |
| Age (year) | 53 ± 18 | 51 ± 14 |
| Females | 44 (70) | 189 (58) |
| Diabetes mellitus | 3 (5) | 23 (7) |
| Smoking status |  |  |
| Never | 27 (43) | 126 (39) |
| Former | 26 (41) | 159 (49) |
| Current | 10 (16) | 42 (13) |
| History of CVD |  |  |
| CAD | 5 (8) | 19 (6) |
| CeVD | 3 (5) | 11 (3) |
| PAI | 2 (3) | 10 (3) |
| >1 location | 0 (0) | 9 (3) |
| Antihypertensive treatment | 19 (30) | 79 (24) |
| Lipid-lowering treatment | 19 (30) | 108 (33) |
| Antithrombotic therapy | 15 (24) | 59 (18) |
| Glucose-lowering therapy | 2 (3) | 19 (6) |
| BMI (kg/m²) | 26.1 ± 4.8 | 26.2 ± 4.8 |
| SBP (mmHg) | 135 ± 18 | 132 ± 19 |
| DBP (mmHg) | 77 ± 10 | 76 ± 9 |
| Total cholesterol (mmol/L) | 5.0 ± 1.0 | 5.1 ± 1.2 |
| LDL-cholesterol (mmol/L) | 2.8 ± 0.9 | 3.0 ± 1.0 |
| HDL-cholesterol (mmol/L) | 1.5 ± 0.4 | 1.5 ± 0.4 |
| eGFR (mL/min/1.73m^2^) | 95 ± 21 | 96 ± 17 |
| Pulse pressure (mmHg) | 58 ± 14 | 56 ± 14 |
| AIx (%) | 29 ± 14 | 30 ± 15 |
| Carotid IMT (**μm)** | 720 [575;875] | 719 [609;821] |

Data are presented as numbers (%), mean ± standard deviation, or median [interquartile range]. Abbreviations: CVD: cardiovascular disease; CAD: coronary artery disease; CeVD: cerebrovascular disease; PAI: peripheral artery disease with arterial interventions; SBP: systolic blood pressure; BMI: body mass index; LDL: low-density lipoprotein; HDL: high-density lipoprotein; eGFR: estimated glomerular filtration rate with the CKD-EPI formula; AIx: augmentation index; IMT: intima-media thickness. *Normal and high cfPWV values are based on the comparison with the age-expected reference values according to healthy references.

| Table S4: Sensitivity analysis for the relationship between arterial stiffness and risk of cardiovascular events in patients with two pathogenic ABCC6 variants | |
| --- | --- |
| Events/ N total | 35/310 |
| Pulse wave velocity (+ 1 m/s)* |  |
| Crude model | 1.15 [1.06;1.24] |
| Model 1 | 1.21 [1.00;1.47] |
| Model 2 | 1.27 [1.04;1.56] |
| Augmentation index (+ 10%) |  |
| Crude model | 1.50 [1.14;1.96] |
| Model 1 | 1.35 [0.97;1.88] |
| Model 2 | 1.47 [1.04;2.08] |

*Hazard ratios are estimated at a mean age of 51 years. Model 1 was adjusted for age and sex. Model 2 was adjusted for age, sex, DM, eGFR, smoking, SBP, antihypertensive therapy, and CV history. Model 2 for PWV was additionally adjusted for the interaction between age and PWV. Abbreviations: py, person-years.

| Table S5: Relationship between arterial stiffness and risk of acute cardiovascular events | |
| --- | --- |
| Events/ N total | 40/390 |
| Pulse wave velocity (+ 1 m/s)* |  |
| Crude model | 1.16 [1.07;1.25] |
| Model 1 | 1.21 [1.01;1.45] |
| Model 2 | 1.26 [1.03;1.54] |
| Augmentation index (+ 10%) |  |
| Crude model | 1.58 [1.23;2.04] |
| Model 1 | 1.50 [1.11;2.01] |
| Model 2 | 1.65 [1.22;2.22] |

*Hazard ratios are estimated at a mean age of 51 years. Model 1 was adjusted for age and sex. Model 2 was adjusted for age, sex, DM, eGFR, smoking, SBP, antihypertensive therapy, and CV history. Model 2 for PWV was additionally adjusted for the interaction between age and PWV. Abbreviations: py, person-years.

## FIGURES


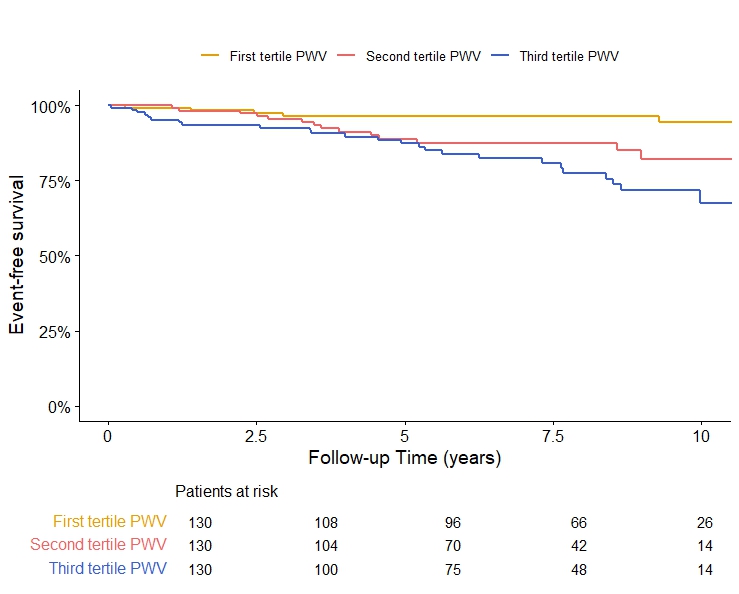


Figure S1: Kaplan-Meier curves for CV-event-free survival according to cfPWV tertiles


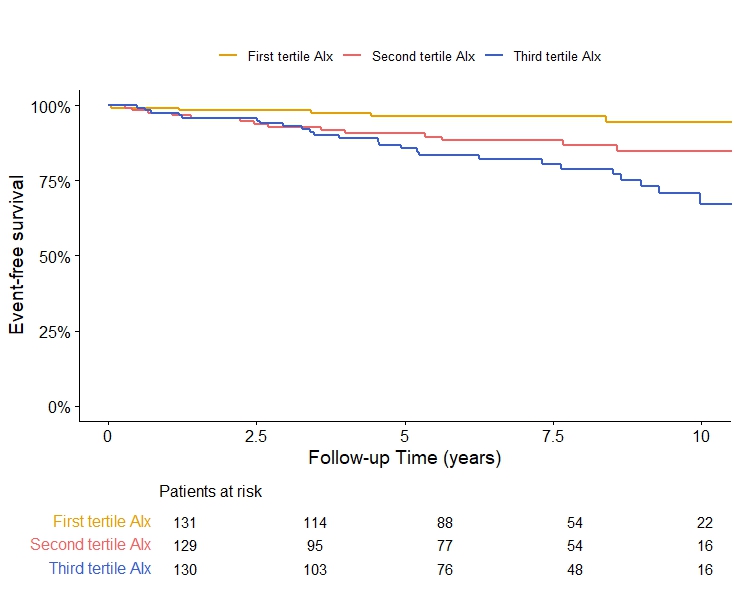


Figure S2: Kaplan-Meier curves for CV-event-free survival according to AIx tertiles
